# Vertical and Horizontal Corneal Epithelial Thickness Profile Using Ultra-High Resolution and Long Scan Depth Optical Coherence Tomography

**DOI:** 10.1371/journal.pone.0097962

**Published:** 2014-05-20

**Authors:** Shuangqing Wu, Aizhu Tao, Hong Jiang, Zhe Xu, Victor Perez, Jianhua Wang

**Affiliations:** 1 Department of Ophthalmology Red-Cross Hospital, Hangzhou, Zhejiang, China; 2 Bascom Palmer Eye Institute, Miller School of Medicine, University of Miami, Miami, Florida, United States of America; 3 School of Ophthalmology and Optometry, Wenzhou Medical College, Wenzhou, China; Duke University, United States of America

## Abstract

**Purpose:**

To determine the vertical and horizontal thickness profiles of the corneal epithelium in vivo using ultra-long scan depth and ultra-high resolution spectral domain optical coherence tomography (SD-OCT).

**Methods:**

A SD-OCT was developed with an axial resolution of ∼3.3 µm in tissue and an extended scan depth. Forty-two eyes of 21 subjects were imaged twice. The entire horizontal and vertical corneal epithelial thickness profiles were evaluated. The coefficient of repeatability (CoR) and intraclass correlation (ICC) of the tests and interobserver variability were analyzed.

**Results:**

The full width of the horizontal epithelium was detected, whereas part of the superior epithelium was not shown for the covered super eyelid. The mean central epithelial corneal thickness was 52.0±3.2 µm for the first measurement and 52.3±3.4 µm for the second measurement (*P*>.05). In the central zone (0–3.0 mm), the paracentral zones (3.0–6.0 mm) and the peripheral zones (6.0–10.0 mm), the mean epithelial thickness ranged from 51 to 53 µm, 52 to 57 µm, and 58 to 72 µm, respectively. There was no difference between the two tests at both meridians and in the right and left eyes (*P*>.05). The ICCs of the two tests ranged from 0.70 to 0.97 and the CoRs ranged from 2.5 µm to 7.8 µm from the center to the periphery, corresponding to 5.6% to 10.6% (CoR%). The ICCs of the two observers ranged from 0.72 to 0.93 and the CoRs ranged from 4.5 µm to 10.4 µm from the center to the periphery, corresponding to 8.7% to 15.2% (CoR%).

**Conclusions:**

This study demonstrated good repeatability of ultra-high resolution and long scan depth SD-OCT to evaluate the entire thickness profiles of the corneal epithelium. The epithelial thickness increases from the center toward the limbus.

## Introduction

The corneal epithelium is the outermost layer of the cornea. It plays an important role in maintaining corneal surface smoothness and corneal power. The corneal epithelial thickness is altered in some corneal diseases such as keratoconus, dry eye, and refractive regression after laser surgery [1], [2], [3]. Analyzing corneal epithelial thickness would facilitate disease diagnosis and management [1]. Optical coherence tomography (OCT) has been used for measuring corneal epithelial thickness and has the advantage of being non-contacting and having good repeatability and safety [4]. Although previous studies imaged the corneal epithelial thickness profiles in single scans, only the central 6-mm zone profiles were acquired because of the limitation of the scan depth [2], [5]. Multiple images at different locations were imaged with OCT to acquire a larger field of the corneal epithelial thickness profiles up to a 10-mm zone by stitching the images. The operation and analysis of the joined images were complicated and time-consuming, and no landmarks could be used for precise registration of these images [6], [7], [8], [9]. Using our newly developed spectral domain OCT (SD-OCT) instrument with an ultra-long scan depth, imaging the entire epithelial profile has been demonstrated using single scans [10]. The resolution of the system could be improved. The goal of this study was to determine the entire epithelium thickness profile in the vertical and horizontal meridians, using ultra-high resolution and ultra-long scan depth SD-OCT.

## Methods

Twenty-one normal subjects with 42 eyes were included. There were 12 females and 9 males, with a mean age of 26.2±4.3 years. No subject had ocular or systemic diseases. None of them use contact lens or any medications. The tear battery tests of each subject were conducted in the present study. They were considered as normal if they did not meet the dry eye diagnosis criterion as follows: one or more dry eye questionnaire symptom is often or always; Schirmer's test score <10 mm; tear break-up time <10 second; corneal fluorescent staining score >1. The study was approved by the University of Miami review board. All of the subjects were treated in accordance with the tenets of the Declaration of Helsinki, and written informed consent was obtained from each subject.

### Custom spectral-domain optical coherence topography

To image the entire cornea, an ultra-long and ultra-high resolution SD-OCT was custom developed. The axial resolution of the SD-OCT system is 4.6 µm in air and 3.3 µm in tissue, with an extended scan depth of 7.281 mm. The scan width was set to 13.465 mm. The SD-OCT was improved from our previous system [10] by using a newly designed spectrometer and a broad band light source. A superluminescent diode light source (Broadlighter, T840-HP, Superlumdiodes, Ltd., Moscow, Russia) with a center wavelength of 840 nm and a full width at half maximum bandwidths of 100 nm was used to provide low coherence light. Coupled with a fiber-based Michelson interferometer, the light passing through a fiber-pigtailed isolator was split into reference and sample arms by a 50:50 fiber coupler. The spectrometer was based on a fast complementary metal-oxide-semiconductor (CMOS) camera (Basler sprint spL4096-140k; 4096 pixels, Basler AG, Germany), an achromatic imaging lens (f = 150 mm), and a 1,800-line/mm transmission grating (Wasatch Photonics, Logan, UT). The power of the incident light was 1.307 mW, which was safe to the eye. A telecentric light delivery system, which was driven by an X-Y galvanometer scanner design and a video viewing system, was co-axially aligned and mounted with a modified slit-lamp biomicroscope. The images acquired by the camera were delivered to a computer workstation for displaying and processing. The system worked at a scan speed of 35,000 axial scans per second.

### Experimental procedure and image analysis

All of the subjects were imaged twice with 16 lines radically by SD-OCT, with 2,048×4,096 pixels. The entire scan pattern was acquired in 1.9 seconds. All the subjects were asked to look at the fixation light and open the eye as widely as possible without having the upper lid lifted by the operator, to reduce the movement of the eyeballs. To decrease the effect of tear film thickness in the epithelial thickness, all OCT scans were performed immediately after blink. Custom software (J-OCT-1, version 1.0) was used to process the epithelial thickness profile by a single investigator (SW), as described in a previous study [9]. According to the Snell principle, a custom algorithm correcting optical distortion was used to remove the image distortion induced by refraction and transition of the group index at the air-epithelium interface [9]. A refractive index of 1.389 was applied [9], [11], [12]. After optical distortion was corrected [9], the epithelial thickness was measured as the perpendicular distance between the front surface and the corrected back surface at each lateral location. The corneal epithelial thicknesses in the horizontal 10-mm zone and the vertical meridian in the 9-mm zone were analyzed at 0.5-mm steps with an average matrix. Two observers (SW and AT) analyzed the horizontal images of the right eyes in the first examination to test the interobserver variability.

### Statistical analysis

The statistical analysis was performed using the SPSS 16.0 software package (SPSS Inc., Chicago, Illinois). The horizontal meridian of the left eye was mirrored to the right eye when compared or the profiles of both eyes were averaged. A paired-*t* test was used to assess the agreement between the two measurements. The coefficients of repeatability (CoR) and the intraclass correlation coefficients (ICC) of the two tests were calculated. The CoR was defined as two standard deviations of the difference between two measurements. The CoR% was the percentage of CoR divided by total mean. All the tests were two-tailed, and *P*<.05 was considered statistically significant.

## Results

**Figure 1:**
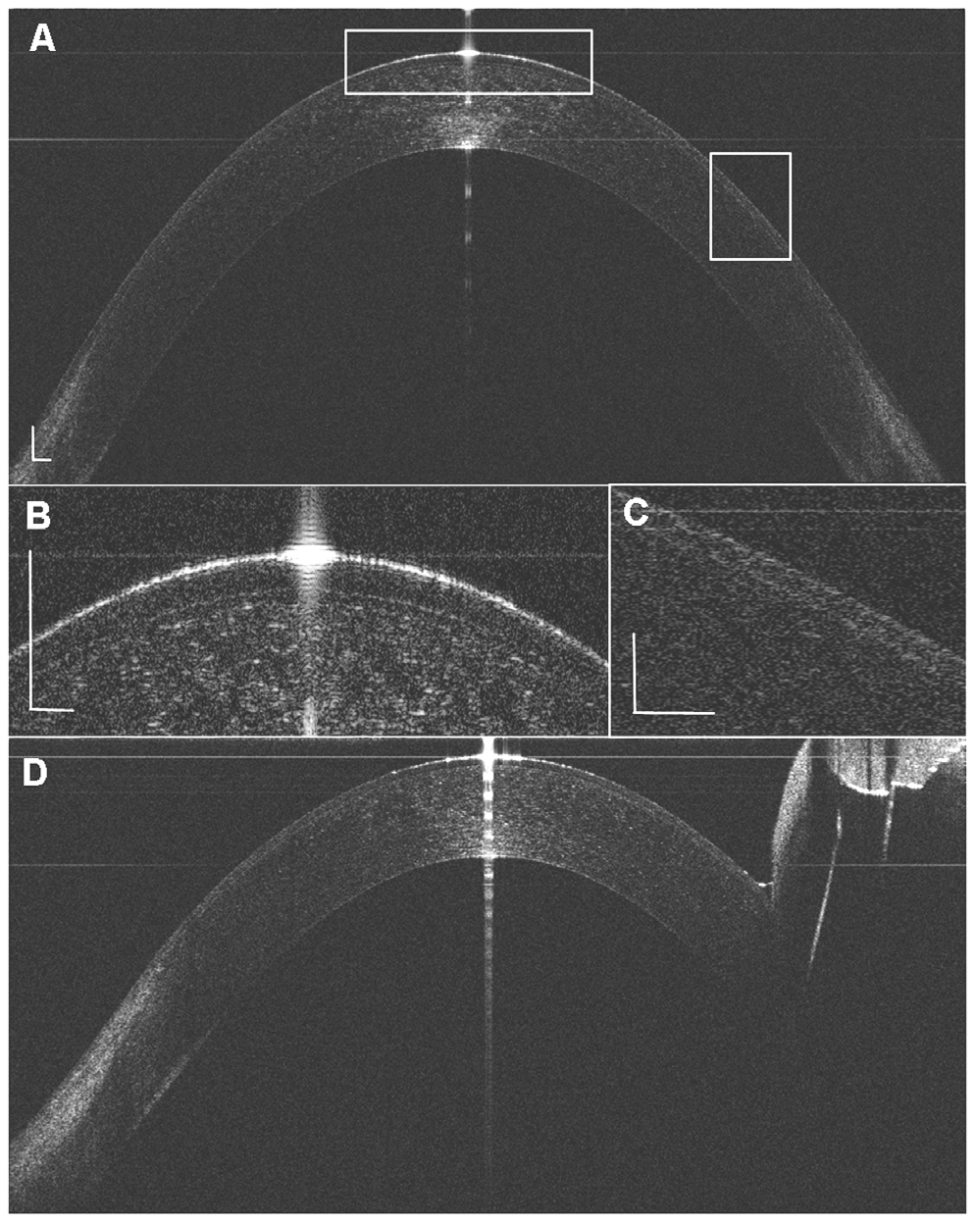
Representative images of the corneal epithelium with ultra-long and ultra-high resolution SD-OCT. (A) The horizontal corneal epithelial thickness image. (B, C) The entire corneal epithelial layer could be distinguished. (D) In the vertical corneal epithelial thickness image, part of the superior cornea was covered by the eyelid, and the tear menisci could be observed clearly (Bar  = 250 µm).

**Figure 2:**
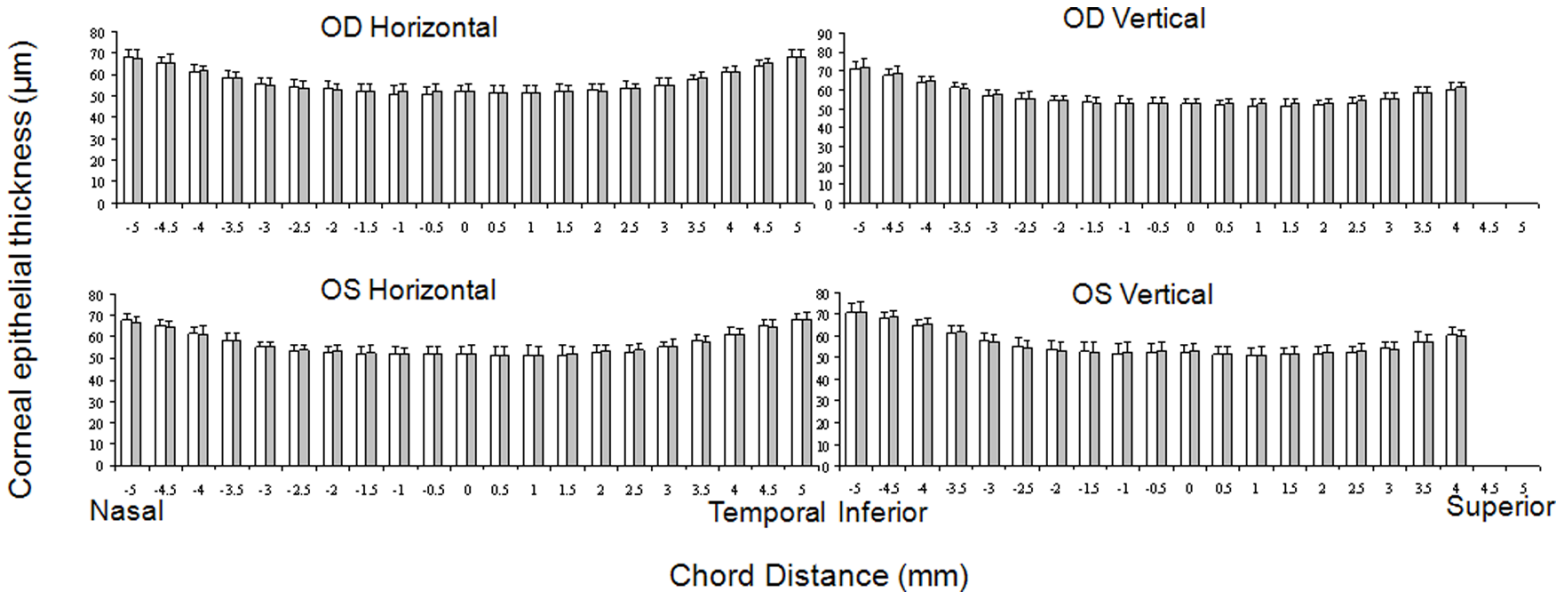
Repeated two tests for the corneal epithelial thickness profiles. There was no significant difference between the repeated Test 1(white bar) and Test 2 (gray bar) with ultra-long and ultra-high resolution SD-OCT in horizontal or vertical meridian in the bilateral eyes (*P*>.05).

The 10 mm width of the horizontal (Figure 1) and the 9 mm width of the vertical corneal epithelium were detected, whereas a part of the superior corneal epithelium was not shown because of the coverage of the superior eyelid. The mean central corneal thickness was 52.0±3.2 µm for the first measurement and 52.3±3.4 µm for the second measurement (*P*>.05). In the central zone (0- to 3.0-mm), the paracentral zones (3.0- to 6.0-mm) and the peripheral zones (6.0- to 10.0-mm), the mean corneal epithelial thickness ranged from 51 to 53 µm, 52 to 57 µm, and 58 to 72 µm, respectively. The corneal epithelium thickness increased significantly in the points from the 3-mm to 5-mm zone compared to the apex point (*P*<.05). The corneal epithelial thickness was similar at all the symmetrical temporal and nasal corneal points (*P*>.05), whereas the epithelium on the inferior points was significantly thicker than that on the symmetrical superior points from the 2.5-mm point (*P*<.05). There was no difference between the repeated two tests in the same eye (*P*>.05) (Figure 2). The ICC of the two tests ranged from 0.72 to 0.97, and the CoR ranged from 2.9 µm to 7.6 µm, corresponding to 5.7% to 10.4% (CoR%).

**Figure 3:**
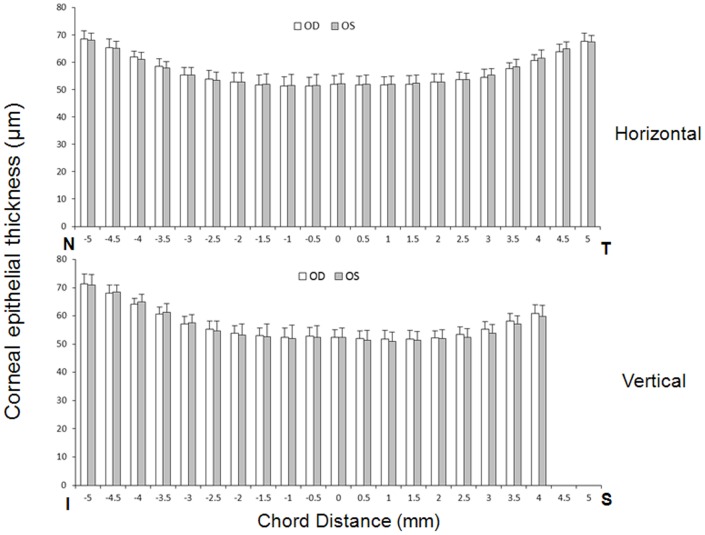
Comparison of corneal epithelial thickness in the bilateral eyes in horizontal and vertical meridian. The horizontal and vertical profiles with ultra-long and ultra-high resolution SD-OCT showed no significant differences between the right eyes and the mirrored left eyes (*P*>.05). The temporal and nasal corneal epithelial thickness increased symmetrically, and the inferior corneal epithelial thickness increased more rapidly than the superior ones. T: temporal; N: nasal; I: inferior; S: superior.

**Figure 4:**
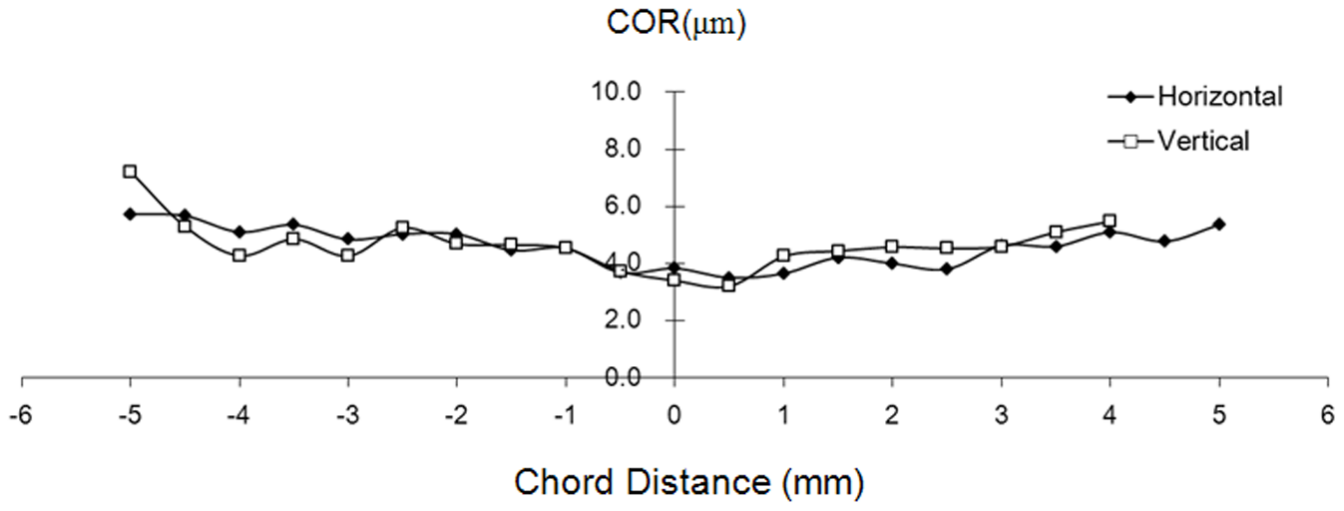
Coefficients of repeatability (CoR) of the horizontal and vertical corneal epithelial thickness profiles. The average CoR of the horizontal and vertical corneal epithelial thickness profiles of the bilateral eyes with ultra-long and ultra-high resolution SD-OCT.

**Figure 5:**
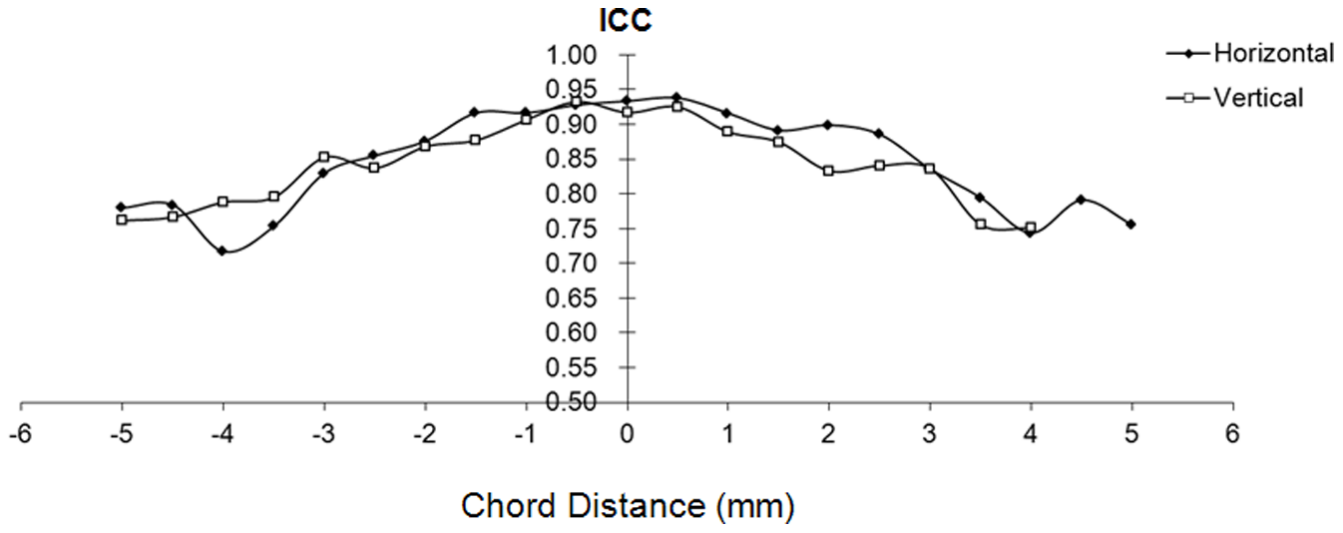
Intraclass correlation coefficients (ICC) of the horizontal and vertical corneal epithelial thickness profiles. The average ICC of the horizontal and vertical corneal epithelial thickness profiles of the bilateral eyes with ultra-long and ultra-high resolution SD-OCT.

The average corneal epithelial thickness of the two tests was not significantly different between the right eye and the mirrored left eye in the horizontal or vertical meridian (*P*>.05) (Figure 3). The average CoR of both eyes in the horizontal and vertical meridians was 4.3±0.54 µm in the central 6-mm zone and 5.3±0.69 µm in the peripheral 4-mm zone (Figure 4). The average ICC of both eyes in the horizontal and vertical meridian decreased from the center to the edge, which was 88%±3.6% in the central 6-mm zone and 77%±2.3% in the peripheral 4-mm zone, respectively (Figure 5).

**Figure 6:**
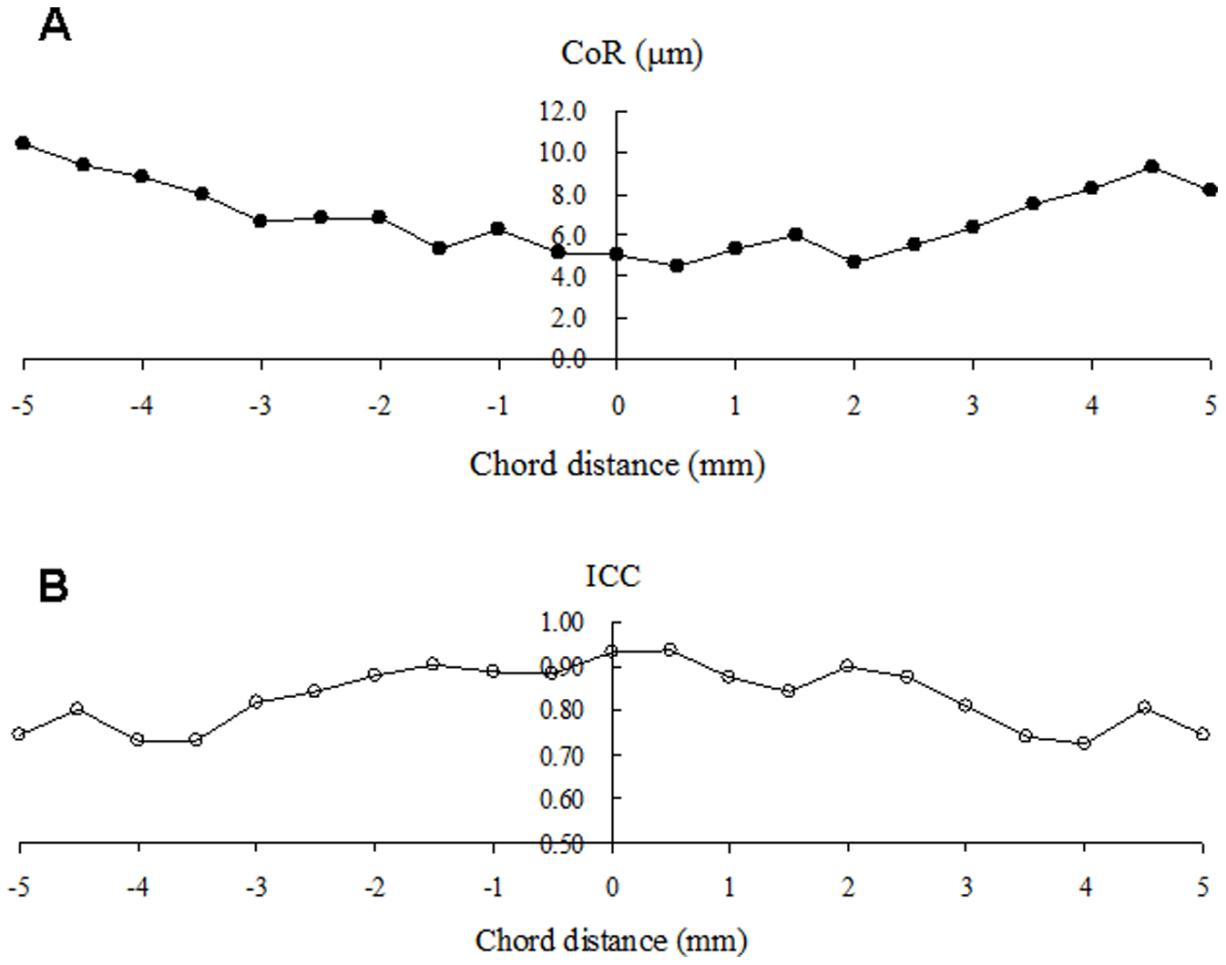
Coefficients of repeatability (CoR) and intraclass correlation coefficients (ICC) between two observers. The horizontal images of the right eyes (n = 21) in the first examination were analyzed by two observers to test the interobserver variability.

The CoRs of the two observers ranged from 4.5 µm to 10.4 µm from the center to the periphery, corresponding to 8.7% to 15.2% (CoR%)(Figure 6 A). The ICC of the two tests by two observers ranged from 0.72 to 0.93 (Figure 6 B). There were no significant differences of epithelial thickness between the two observers at all locations (*P*>.05).

## Discussion

The corneal epithelium is an important layer for corneal surface smoothness and corneal power. Precisely imaging the epithelial thickness is important for disease diagnosis, prognosis and follow-up. Because of the technical limitations of current clinical imaging modalities, it is predominantly the central epithelium that has been imaged, although some attempts have been made to image the epithelial profile and map the limited corneal areas. The alternation of the corneal epithelium has been studied in ocular diseases and conditions, and precise measurements of the layer are critical for disease diagnosis, staging, and prognosis, as well as for follow-up. Contact lens [5], [6], [7], [10], refractive surgery [13], [14], [15], [16], dry eye [3], [17], and keratoconus [2], [5], [18] have been found to cause changes in corneal epithelial thickness. Wilson et al. [19] measured the thickness of the corneal epithelium with micropachymetry. High frequency ultrasonic pachymetry [14], [20] and confocal microscopy [21] were used to measure corneal epithelial thickness. These examinations are invasive methods requiring contact with the eye. OCT, a noninvasive and non-contact optical imaging technique, acquires corneal epithelial images.

Most of the OCT-related studies measured the corneal profiles in the central cornea [13], [16], [22], [23] or the peripheral profile in the horizontal meridian [6], [7], [8], [10] or mapped a center region [2], [3], [5], [13], [15]. A few studies elucidated the entire corneal thickness profile in the vertical meridian [5], [9], [24]. Commercial OCT could image the central 6-mm corneal epithelial thickness. Although the imaging width could cover the entire cornea by a modified fixation target and multiple scans at different locations, the examination and analysis were time-consuming, and registering the entire epithelium might be difficult. An ultra-long scan depth SD-OCT could be used to acquire entire corneal epithelial profiles in a single scan. We recently reported the corneal epithelium and contact lens profiles in the horizontal meridian [10]. In this study, improved resolution of the ultra-long scan depth SD-OCT from 5.1 µm to 3.3 µm might be beneficial, and a test of the repeatability for the entire corneal epithelial thickness might be essential for further study designs using the imaging modality.

Investigating the extent of repeatability is important for evaluating new capability of imaging devices to measure the entire epithelial thickness in clinical applications. It might help in determining sample sizes in future clinical studies. The CoR and CoR% reflected the repeatability of two examinations, and ICC indicated the agreement between two examinations. Low CoR values and high ICC values indicate the excellent repeatability. To the best of our knowledge, this study was the first to determine the entire epithelial thickness profile using a long scan depth and ultra-high resolution SD-OCT. We found that the corneal epithelial thickness could be detected reliably with this new SD-OCT. The repeatability obtained with SD-OCT appeared to be much better than the results obtained with the time-domain OCT [13], [25]. The CoR in the central cornea ranged from 3.2 µm to 5.2 µm in the 6-mm zone, which was consistent with the resolution of the instrument. This central CoR is similar to the repeatability of our previous ultra-long scan depth SD-OCT (3.0 to 6.0 µm) [10] and comparable to Prakash et al's result [26], but slightly worse than the reported 1-mm central CoR in a previous study of three OCT instruments [16]. Several studies [2], [3], [15] with the commercial RTVue reported 6-mm central CoR with pooled standard deviation, which was range from 0.7 to 1.9 µm, implying a different study design and image analysis correlated with variation of CoR. The present CoR ranged from 4.6 µm to 7.2 µm in periphery cornea, which is identical to the results of Tao et al. (6.6 µm to 8.2 µm) [10]. In this study, with the long scan depth of our system, the entire corneal profiles could be acquired with one scan. As expected, the periphery CoR decreased, which might be because of the signal-to-noise ratio (SNR) drop with an increase of depth in the SD-OCT. In this study, the central 6-mm ICC value was 88%, and the periphery 4-mm was 77%. The central 2-mm ICC value was >91%, in agreement with that of the studies of Ge et al. and Ma et al [15], [16]. The decreasing ICC value from central to limbal in our instruments was explainable with the decreasing SNR. Using the phase shift might compensate for the drawback of the SNR drop. The implementation of the phase shift is currently under development [27].

We found the percentage of CoR between observers was approximately 4.5 µm at the center, 5.0 to 6.8 µm within 3 mm range, and 7.4 to 10.4 µm at the limbus. The ICC of the two tests by two observers ranged from 0.72 to 0.93. The interobserver variability in the present study was slightly worse than the intraobserver variability but they are comparable. Lian et al [24] also reported good repeatability (CoR range 0.8 to 1.2 µm) in repeated images by a single observer, implying intraobserver repeatability was more reliable.

Our results obtained with the new imaging modality are in agreement with the measurements previously reported [2], [5], [8], [9], [10], [17], [24],[28]. We detected central corneal epithelial thickness (the apex) of approximately 52 µm, which is consistent with our previous studies [8], [9], [10], [24] and in the range of other studies (Table 1). Most studies reported meridian profiles or a map in a 6-mm zone (Table 1). The horizontal meridian appeared to increase slightly from the center to the limbus, and the trend was consistent in a 10-mm zone [2], [5], [8], [9], [10], [17], [24], [28]. Our horizontal results are in agreement with these studies. The results of the vertical meridian profiles appeared to vary in previous OCT studies, and a limited number of studies reported the results in a 10-mm zone [9], [24]. Reinstein et al. [20] found with high-frequency digital ultrasound that the corneal epithelium was thin in the center and thicker inferiorly than superiorly in the normal corneas. The corneal epithelium thickened from the center to the limbus, and the epithelial thickness increased more gradually in the superior region than in the inferior region, reaching statistical significance at the 2.5-mm chord distance, which was a trend similar to the results of Reinstein et al. In a previous study, Haque et al. [5] reported a reversed trend of the vertical meridian profile with a commercial time-domain OCT in an 8-mm zone. Our previous studies investigated the corneal epithelial thickness in the vertical meridian in a ∼12-mm zone with three segmentations and detected that the superior epithelial thickness was less than the inferior epithelial thickness [9], [24]. These differences were most likely because of the distinctive detection and definition of the epithelial zone. One scan to cover the entire profile might be preferable to the stitching of multiple scans because there are no landmarks for registration. Stitching multiple images might induce more measurement errors.

**Table 1:** Normal Corneal epithelial thickness profiles imaged with OCT.

Year	Author	OCT Type	Resolution (µm)	Depth (mm)	Profiles	Width (mm)	Central ET(µm)	CoR (µm)	ICC
Present	Wu et al.	custom SD-OCT	3.3	7.28	Two meridians	10	52	3.2 to 7.2	72% to 97%
2014	Kanellopoulos et al.[3]	RTVue SD-OCT	5	1.96	Map	6	53.0±2.7	NA	NA
2013	Tao et al.[10]	custom SD-OCT	5.1	10.4	Horizontal meridian	10	51.9±3.5	1.5 to 4.1^*^	NA
2013	Rocha et al.[18]	RTVue SD-OCT	5	1.96	Two meridians	5	50.5±3.9	NA	NA
2013	Lian et al. [24]	custom SD-OCT	3	2	Two meridians	>10	52	NA	NA
2013	Ma et al. [15]	RTVue SD-OCT	5	1.96	Map	6	53.2	0.7 to 1.2^**^	>92%
2013	Ge et al.[16]	custom SD-OCT	3	1.85	Horizontal meridian	1	54.0±2.0	1.2	95%
		custom SD-OCT	7.5	5.6	Horizontal meridian	1	55.1±2.3	2.1	90%
		RTVue SD-OCT with modified lens	5	1.96	Map	1	53.8±2.3	2.2	89%
2012	Schmoll et al.[29]	SD-OCT	1.3	2.7	Map	6	56.7±3.7	NA	NA
2012	Du et al.[9]	custom-built SD-OCT	3	1.85	Two meridians	>10	52.5±2.4	NA	NA
2012	Li et al.[2]	RTVue SD-OCT	5	1.96	Map	6	52.3±3.6	0.7 to 1.9^**^	NA
2012	Prakash et al. [26]	Cirrus HD SD-OCT	5	2	Horizontal meridian	3	58.6±4.2	4.5	93%
2011	Tao et al.[8]	custom-built SD-OCT	3	3	Horizontal meridian	>10	52.5±2.4	NA	NA
2011	Francoz et al.[17]	SD-OCT modified with anterior segment module	3.9	1.8	Horizontal meridian	2–3	48.3±2.9	NA	NA
2010	Hutchings et al.[28]	Custom SD-OCT system	3.2	1	Horizontal meridian	5	58.4±1.0	NA	NA
2008	Haque et al.[5]	Zeiss TD-OCT with modified fixation	13	2.7	Four meridians and map	8	53.6±2.2	NA	NA
2006	Sin et al.[13]	Zeiss TD-OCT	13	2.7	Horizontal meridian	1.13	52±3	12.8^**^	>36%
2002	Wang et al.[25]	Zeiss TD-OCT with modified fixation	13	2.7	Horizontal meridian	10	57.8±1.7	2.5^*^	NA
2001	Feng et al.[23]	Zeiss TD-OCT	13	2.7	Horizontal meridian	1	61.2±2.3	NA	NA
1994	Izatt et al.[22]	Custom TD-OCT	10	NA	Horizontal meridian	NA	81	NA	NA

OCT  =  optical coherence tomography; SD  =  spectral domain; TD  =  time domain; ET  =  epithelial thickness; CoR  =  coefficients of repeatability; ICC  =  intraclass correlation coefficients; NA  =  not available.

CoR was reported as two standard deviations of the difference between two measurements.^ *^CoR was reported as one standard deviation of the differences between two measurements.^ **^CoR was defined as pooled standard deviations of the difference between two measurements.^ #^CoR was concerning about the repeatability of two repeated images in a single measurement.

There were some limitations in this study. First, the focus of this study was to determine the horizontal and vertical epithelial thickness profiles by testing the repeatability. We did not lift the upper lid, which avoided disturbing the eyeball and movement. The imaging protocol resulted in an incomplete scan of the thickness profile at the vertical meridian. Second, to avoid the effect of corneal edema on the measurement of epithelial thickness, all the subjects were assessed during 10:00 am to 4:00 pm. However, there may be some physiology variation in epithelial thickness because the tear film was included in the measurement. Third, we only investigated normal subjects to demonstrate the imaging capability. Imaging of eyes with diseases such as keratoconus or after a LASIK procedure might have different levels of precision. Further studies are needed concerning these eye diseases. Fourth, we used semi-automatic software to process the images, which was difficult and inclined to product bias. Further improvement could be made by developing automated segmentation, which might improve the measurement precision. Fifth, we only imaged the two meridians. The reconstruction of three-dimensional thickness maps of the entire epithelial should be the next development in the field. Further improvement of the scan speed and the implementation of SNR enhancement such as the phase shift technique might realize the goal.

In summary, we demonstrated ultra-high resolution and long scan depth SD-OCT for the measurement of the entire epithelium thickness profile with good repeatability. The epithelial thickness increases from the center toward the limbus. The approach would be useful for future research and clinical studies.
